# Presentation of cerebral and cervical arterial dissections in Botucatu, Brazil: case series

**DOI:** 10.1590/1677-5449.200242

**Published:** 2021-09-17

**Authors:** Gabriel Pinheiro Modolo, Elaine Keiko Fujisao, Niura Aparecida de Moura Ribeiro Padula, Felipe Aranibar Soares da Silva, Gustavo José Luvizutto, Marcone Lima Sobreira, Rodrigo Bazan, Carlos Clayton Macedo de Freitas

**Affiliations:** 1 Universidade Estadual Paulista – UNESP, São Paulo, SP, Brasil.; 2 Universidade Estadual de Campinas – UNICAMP, Faculdade de Ciências Médicas – FCM, Campinas, SP, Brasil.; 3 Faculdade de Medicina de Ribeirão Preto – USP, Ribeirão Preto, SP, Brasil.

**Keywords:** artery dissection, stroke, endovascular treatment, dissecção da artéria, acidente vascular cerebral, tratamento endovascular

## Abstract

Spontaneous dissection of the cervical and cerebral arteries is an important cause of stroke and disability in young patients. In this report, the authors present a case series of patients with spontaneous carotid, vertebral, or cerebral artery dissection who underwent digital angiography. A review of the published literature on this subject is also presented.

## Resumo

A dissecção espontânea das artérias cervicais e cerebrais é uma causa importante de acidente vascular cerebral e incapacidade em pacientes jovens. Neste relato, é apresentada uma série de casos de pacientes com dissecção espontânea da artéria carótida, vertebral ou cerebral submetidos à angiografia digital. Além disso, é fornecida uma revisão da literatura sobre esse assunto.

## INTRODUCTION

Cerebrovascular disease is the leading cause of severe disability and mortality in Brazil.^1^^,^ 2 Spontaneous dissection of the carotid and vertebral arteries is responsible for 2% of all ischemic strokes, with higher occurrence in young people,^3^^,^ 4 affecting their most productive years.^5^^-^ 8

The most common risk factors include arterial hypertension, smoking, dyslipidemia, fibromuscular dysplasia, collagen disorders, trauma, and migraine with aura.^3^^,^ 7 ^,^ 9 ^-^ 11 Sudden onset neck pain associated with nausea, vomiting, and signs of neurological deficits are strong indicators of cervical artery dissection. Dissection leads to formation of a mural hematoma, resulting in vascular stenosis, pseudoaneurysm, and eventual rupture of the vessel.^10^ Although the clinical presentation may indicate a serious condition such as stroke or subarachnoid hemorrhage (SAH), prognosis and recovery are typically positive and most patients achieve functional independence.^5^^-^ 7 In addition, the incidence of recurrent stroke in these patients is usually low.^12^

Diagnosis of cervical or vertebral dissection is based on a combination of clinical and radiological findings. Typically, the vessel is analyzed with computed angiography tomography (CTA) or magnetic resonance angiography (MRA). However, the gold standard is digital subtraction angiography (DSA). This method, although more invasive, allows possible treatment of stenosis and local complications such as pseudoaneurysms. Clinical management of this disease is still a matter of debate. Therefore, the main aim of this study was to trace the epidemiological profile of this disease in our region within the last 5 years.

## PATIENTS AND METHODS

The protocol was approved by the institution Ethics Committee Botucatu Medical School (number 535/2012) and informed consent was obtained. We created a database with information extracted from the Botucatu Stroke Unit electronic medical record. We identified 60 patients with cerebral or cervical arterial dissection who underwent DSA. Eleven patients were excluded due to a lack of information in their medical records or to a different final diagnosis after undergoing DSA. Therefore, 49 patients were included who had been diagnosed with arterial dissection and underwent DSA from January 2013 to December 2018. Demographic information, clinical signs, and symptoms were collected upon admission. In-hospital evaluation and outpatient follow-up were conducted using the National Institute of Health Stroke Scale (NIHSS) and the modified Rankin Scale (mRS). Associated risk factors such as systemic arterial hypertension, smoking, diabetes, and clinical and/or endovascular therapy were also analyzed.

## RESULTS

Of the 49 patients with carotid or vertebral/basilar artery (extra or intracranial) dissection, 42 presented with an ischemic event due to vessel stenosis or arterial thrombus embolism, and 6 presented with SAH due to rupture of the vessel and formation of a dissecting aneurysm. The mean patient age was 55 years, and 51% of the patients were female. Dissection occurred at the carotid level in 31 patients and in the vertebral and basilar arteries or their branches in 18 patients.

Headache and cervical pain were the most common symptoms. In up to one-third of all cases, nausea, vomiting, and coordination deficits were found principally in vertebrobasilar territory events. Additionally, sensory/motor and visual deficits were common. In cases of dissection with pseudoaneurysm, the signs and symptoms were associated with SAH and impaired consciousness.

The most common risk factors included systemic arterial hypertension, smoking, diabetes, and dyslipidemia. Almost 10% of all cases had a prior history of migraine. Hyperhomocysteinemia and hypothyroidism were rare.

Treatment followed the stroke unit protocols. Antiplatelet therapy combined with statin was the most frequent treatment (63.2%). Dual antiplatelet therapy was indicated for 22.4%, anticoagulant combined with statin was administered in 4%, and 46.9% underwent endovascular treatment. Mechanical thrombectomy associated with application of intracranial or extracranial stents in cases of dissection with tandem occlusion was indicated for 5 patients (10.2%) with intracranial occlusion, only 1 of whom received recombinant tissue plasminogen activator. Pure intracranial thrombectomy was performed in 2 patients (4%).

Patients with SAH and pseudoaneurysm formation were treated in the acute phase predominantly with platinum coils and intracranial stents, excluding brain circulation injury, and it was necessary to occlude the vessel as treatment for the pseudoaneurysm in only 1 patient. Most cases had an mRS<2 at discharge. Patient demographic characteristics are shown in Table 1.

**Table 1:** Demographic characteristics of entire sample (n = 49).

**Variables**	**n**	**%**
Age (years)	55 (16-85)	
Patients < 45 years old	12	24.48
Sex (Female)	25	51.02
NIHSS at admission (median)	2 (0-9)	
NIHSS at discharge (median)	0 (0-3.5)	
mRS at admission (median)	0 (0-1)	
mRS at discharge (median)	0 (0-2)	
Arterial hypertension	32	65.30
Smoking	27	55.10
Dyslipidemia	15	30.61
Diabetes	10	20.40
Migraine	5	10.20
Obesity	5	10.20
Previous arterial disease (MI, stroke, or PAD)	10	20.40
**Clinical Presentation**		
Pain (Headache or cervical)	15	30.61
Hemiparesis	12	24.48
Aphasia	2	4.08
Impaired consciousness	8	16.32
Visual impairment	5	10.20
Ataxia	6	12.45
Subarachnoid hemorrhage	6	12.45

NIHSS: National Institute of Health Stroke Scale; mRS: modified Rankin Scale; MI: myocardial infarction; PAD: peripheral artery disease.

The median NIHSS score at discharge was 0 (0–3.5) points, compared to a 2 (0–9) point median score at admission. There were 2 deaths, one due to vertebral dissection and basilar occlusion, and the other due to pseudoaneurysm and SAH. Of the 49 patients included, 61.2% had an mRs<2 (good outcome) after 90 days.

The cervical and intracranial carotid arteries were the most often compromised (60% of all cases). The lesion pattern was stenosis in all cases of cervical carotid, and pseudoaneurysm in most intracranial carotid cases (18 of 19 patients). Cervical lesions had ischemic presentation, and intracranial lesions presented as SAH. Vertebral arteries were affected in almost one-third of all cases. The angiographic findings are shown in Table 2.

**Table 2:** Angiographic findings and treatment modality (n = 49).

**Compromised artery**	**n**	**%**
Cervical carotid	12	24.48
Intracranial carotid	19	38.77
Vertebral	14	28.57
Basilar	1	2.04
Posterior cerebral	2	4.08
Superior cerebellar	1	2.04
**Angiography pattern**		
Pseudoaneurysm	18	36.73
Stenosis	31	63.26
**Treatment**		
Anticoagulation	2	4.08
Antiaggregation	31	63.26
Endovascular	23	46.93

## DISCUSSION

Cerebral and cervical artery dissection are important causes of stroke, especially for people under 45 years, where they may be responsible for approximately one-fifth of all cases.^5^^-^ 7 The mean patient age in our case series was 55 years (range, 16–85), and these findings are probably related due to the selection criteria used for this study. All patients in our institution underwent CTA in the acute stroke phase and were only sent to the angiography suite when there were complications or the diagnosis was dubious. This may also explain the large number of SAH cases in our sample.

The pathophysiology of the disease is related to tears in the arterial endothelium and an intraluminal hematoma that causes delamination of the vessel wall layers, resulting in a false lumen. When cerebral or cervical vessels are affected, a stroke is caused by local growth of the false lumen with occlusion of the vessel, thrombus formation with focal or distal occlusion, compromise of the perforated artery ostium, and hemorrhage due to vessel wall rupture, aneurysm, or pseudoaneurysm formation.

**Figure 1:**
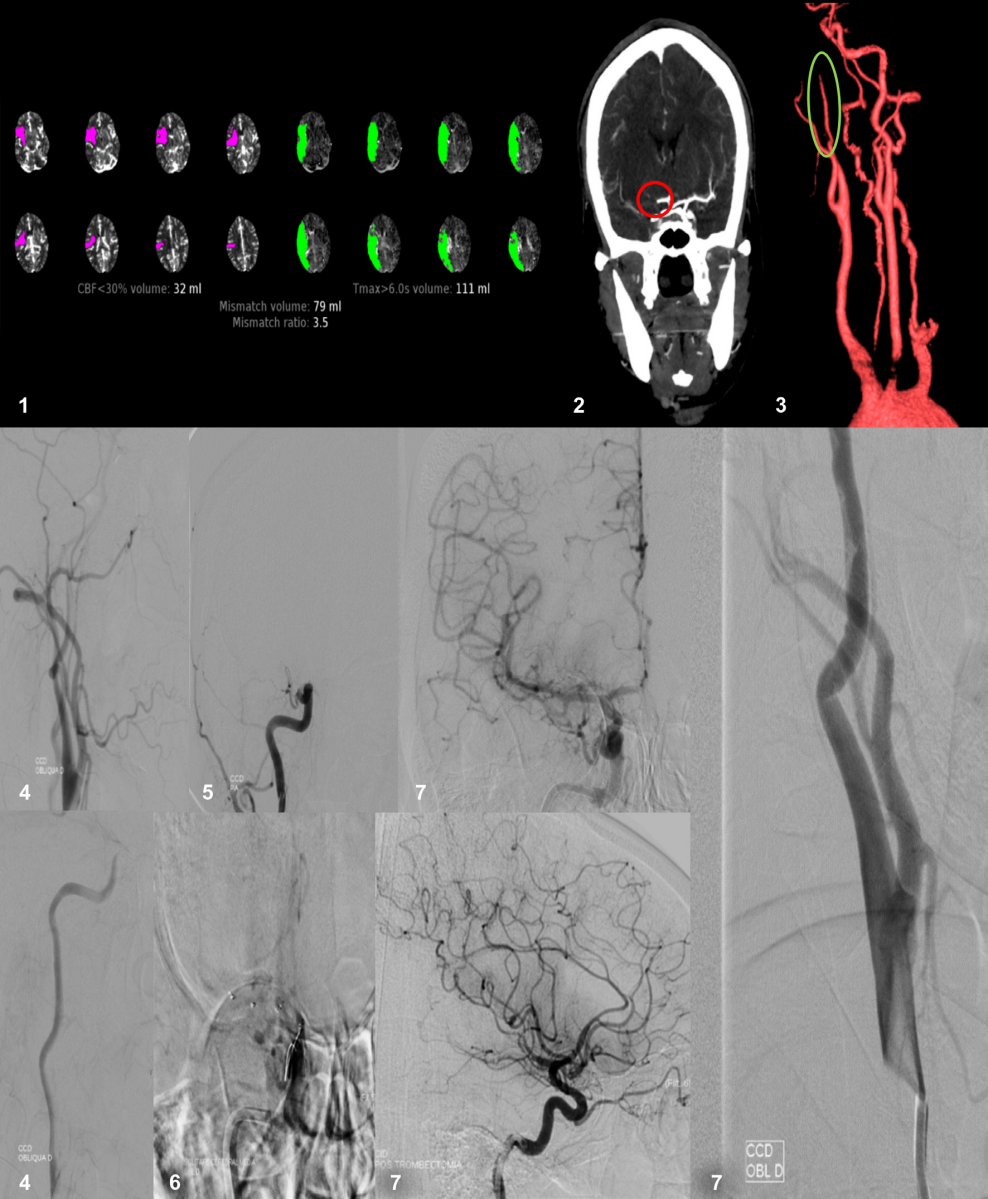
Patient that was admitted with acute stroke due to tandem occlusion and underwent mechanical thrombectomy. 1. Perfusion CT showing an acute stroke with large penumbra. 2. Median cerebral artery occlusion on CTA. 3. Internal carotid dissection with “pencil sign” on 3D CTA. 4. Angiographic image of carotid dissection and retarded contrast flow. 5. Median cerebral artery occlusion on DSA. 6. Position of stent retriever. 7. Post thrombectomy intracranial and cervical carotid images.

Our study results were consistent with the main common risk factors,^3^^,^ 5 ^,^ 11 ^,^ 13 ^-^ 16 showing that most patients had hypertension, dyslipidemia, and were smokers. We found that only 10% of patients had migraine and no patients had hyperhomocysteinemia, but this result may be due to the older patient ages in our study. Based on clinical presentation, many SAH patients were selected for DSA. When the cervical carotid or vertebral territories were compromised, we observed widely described clinical symptoms such as headache, cervicalgia, hemiparesis, visual deficits, vertigo, and ataxia. Ischemic stroke was more common, although this was probably due to the requirement for DSA treatment in our patient sample. Notably, we found many patients presenting with tandem occlusions, as shown in Figure 1.

**Figure 2:**
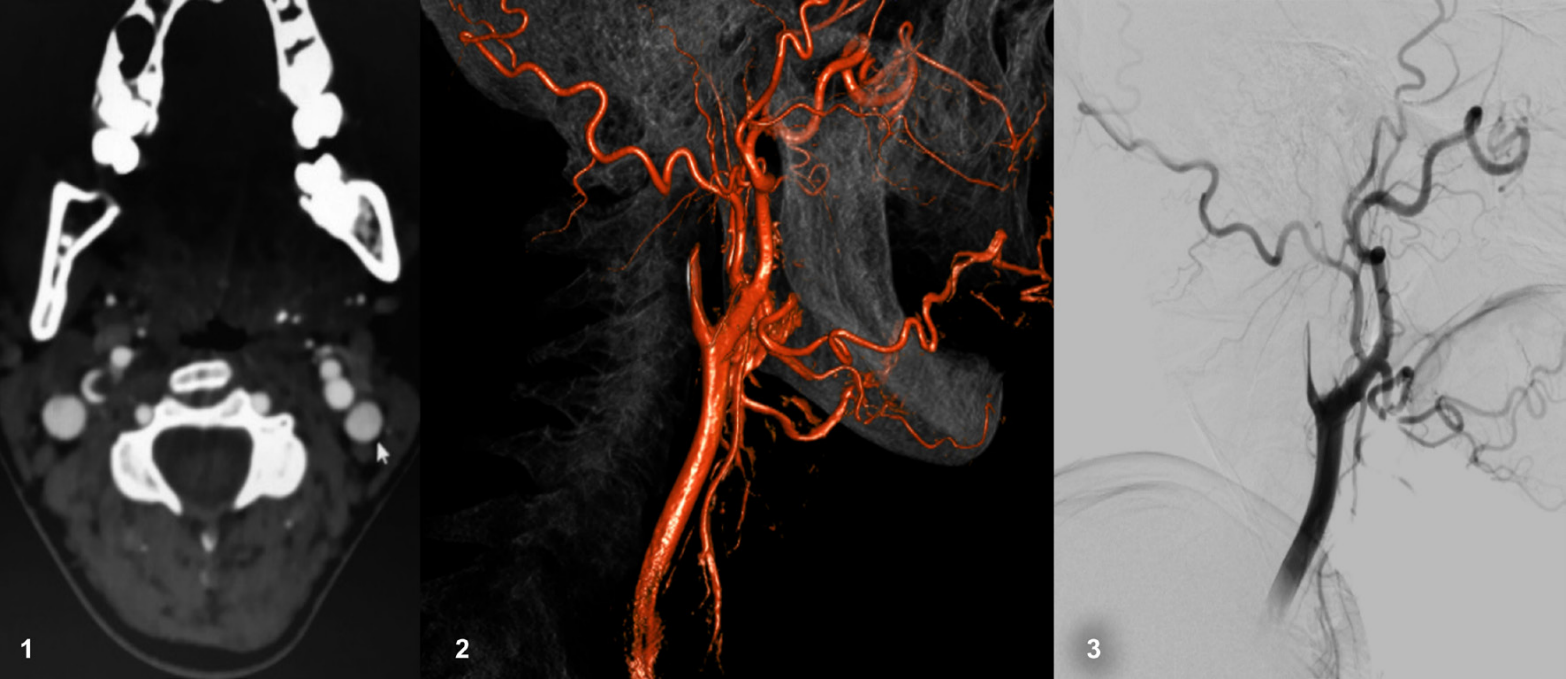
Radiology findings in arterial dissection case. 1. Crescent sign. 2 and 3. Flame shaped carotid occlusion.

**Figure 3:**
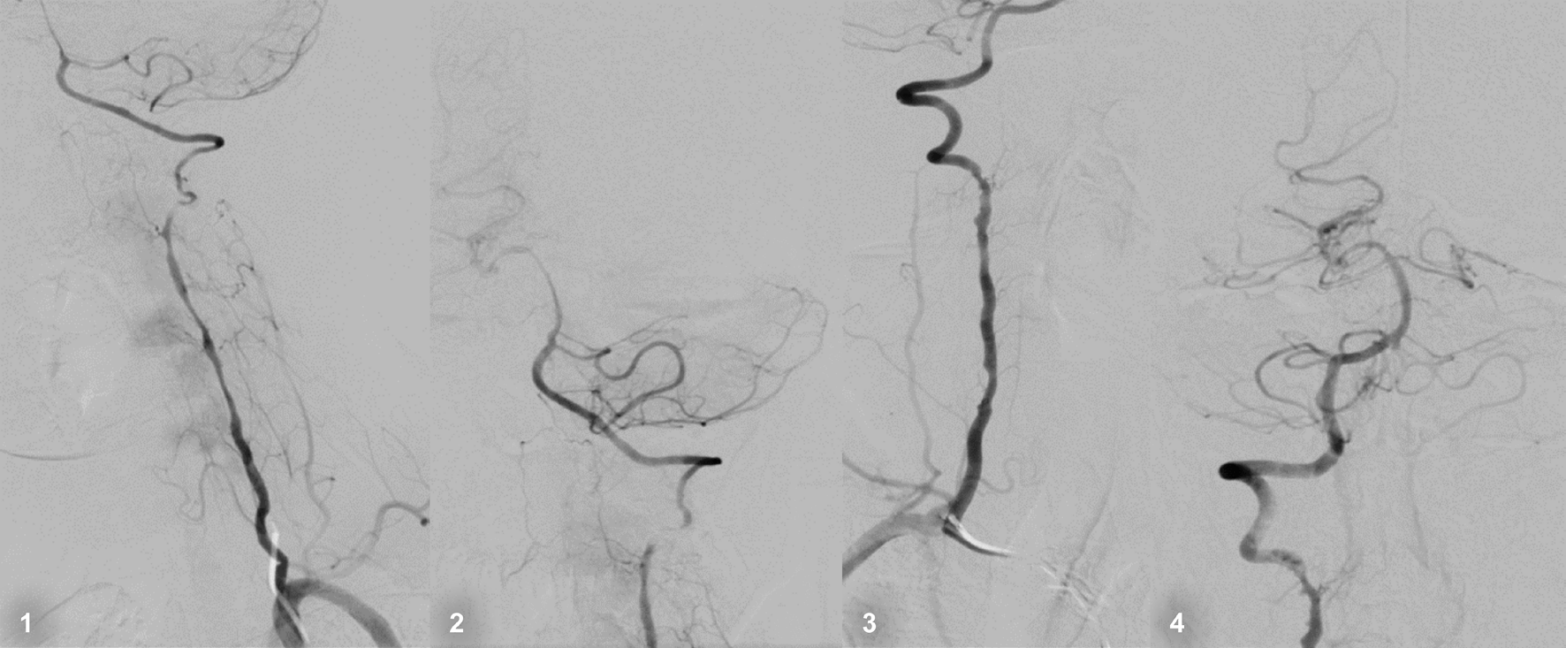
Bilateral spontaneous vertebral artery dissection in a patient with suspected fibrodysplasia.

A diagnosis of cerebral and cervical arterial dissection is typically based on clinical and radiological results. Usually, findings include crescent-shaped thickenings, eccentric narrowed lumens, pencil, rat-tail, or candle-flame shaped occlusions, pseudoaneurysms, and intimal flaps with false lumens (Figure 2). Most patients present with a single vessel lesion. However, patients with vessel wall disease, such as fibrodysplasia, may show multiple lesions and compromised vessels (Figure 3). Lesions are more common in the V3 segment of vertebral arteries, and the cervical segment of the carotid artery. In these cases, the vessel tear tends not to affect the carotid bulb. The disease is typically more frequent in the cervical carotid artery, as we observed in our patient sample.

The treatment approach for cerebral and cervical arterial dissection is currently a matter of debate. A Cochrane systematic review comparing antiaggregants and anticoagulants found no differences in death, stroke recurrence, or functional outcomes.^17^^-^ 19 Based on these findings, many authors prefer to use antiplatelet therapy, since major hemorrhagic complications tend to be less frequent with this treatment.^12^ No consensus exists on dual or single antiaggregant use. In the CADISS trial, administration of single or dual agents was a decision made by the investigator, and consequently the results could not be analyzed separately.^20^

Thrombolysis and thrombectomy may safely be performed in cases of ischemic stroke, and no patient should be denied reperfusion therapies.^21^^,^ 22 Figure 3 illustrates a successful case of mechanical thrombectomy in a patient with acute stroke and cervical carotid dissection. Endovascular treatment of arterial dissection is thought to be safe and may be an option in selected cases, especially when complications such as pseudoaneurysms are found.^23^

The functional prognosis for cerebral and cervical arterial dissections tends to be positive, with most patients achieving functional independence.^5^^,^ 15 ^,^ 17 ^,^ 24 However, cases with extensive infarct areas and SAH may have poor recovery, and the functional outcome is more dependent on cerebral damage than it is on vessel wall compromise. Stroke recurrence is low, and very few patients present with new strokes, regardless of the treatment approach.^3^^,^ 6 ^,^ 12 ^,^ 15 ^,^ 19

## CONCLUSIONS

Cerebral and cervical artery dissections are a common cause of stroke for which potential treatment options exists to prevent poor outcomes. Although sometimes neglected, this disease should be recognized and understood by specialists including neurologists, neuroradiologists, and vascular surgeons.

## References

[1] Brazilian Cerebrovascular Disease Society (2001). Primeiro consenso brasileiro do tratamento da fase aguda do acidente vascular cerebral. Arq Neuropsiquiatr.

[2] Saposnik G, Del Brutto OH, Iberoamerican Society of Cerebrovascular Diseases (2003). Stroke in South America: a systematic review of incidence, prevalence, and stroke subtypes. Stroke.

[3] Pieri A, Spitz M, Valiente RA, Avelar WM, Silva GS, Massaro AR (2007). Dissecção espontânea das artérias carótidas e vertebrais em uma população multiétnica. Arq Neuropsiquiatr.

[4] Griffiths D, Sturm J (2011). Epidemiology and etiology of young stroke. Stroke Res Treat.

[5] Reges DS, Mazzeo M, Rosalino R, Gagliardi VDB, Cerqueira LG, Gagliardi RJ (2019). Cervical arterial dissection: Clinical characteristics in a neurology service in São Paulo, Brazil. Arq Neuropsiquiatr.

[6] Dziewas R, Konrad C, Dräger B (2003). Cervical artery dissection - Clinical features, risk factors, therapy and outcome in 126 patients. J Neurol.

[7] Mergeani A, Popescu D, Antochi F (2010). Spontaneous intracranial internal carotid artery dissection. Rom J Neurol Rev Rom Neurol..

[8] Wang G, Zhang Z, Ayala C, Dunet DO, Fang J, George MG (2014). Costs of hospitalization for stroke patients aged 18-64 years in the United States. J Stroke Cerebrovasc Dis.

[9] Shin DH, Hong JM, Lee JS (2014). Comparison of potential risks between intracranial and extracranial vertebral artery dissections. Eur Neurol.

[10] Pezzini A, Grond-Ginsbach C, Debette S (2008). Genetics of cervical artery dissection. Riv Ital di Neurobiol..

[11] Guidetti D, Rota E, Morelli N, Immovilli P (2014). Migraine and stroke: “vascular” comorbidity. Front Neurol.

[12] Markus HS, Hayter E, Levi C, Feldman A, Venables G, Norris J, CADISS trial investigators (2015). Antiplatelet treatment compared with anticoagulation treatment for cervical artery dissection (CADISS): a randomised trial. Lancet Neurol.

[13] Wolfe CDA, Giroud M, Kolominsky-Rabas P (2000). Variations in stroke incidence and survival in 3 areas of Europe. Stroke.

[14] Touzé E, Gauvrit JY, Moulin T, Meder JF, Bracard S, Mas JL, Multicenter Survey on Natural History of Cervical Artery Dissection (2003). Risk of stroke and recurrent dissection after a cervical artery dissection: a multicenter study. Neurology.

[15] Taylor FR (2007). Incidence and outcome of cervical artery dissection: a population-based study - Commentary. Headache.

[16] Debette S (2014). Pathophysiology and risk factors of cervical artery dissection: what have we learnt from large hospital-based cohorts?. Curr Opin Neurol.

[17] Arnold M, Kappeler L, Georgiadis D (2006). Gender differences in spontaneous cervical artery dissection. Neurology.

[18] Perry BC, Al-Ali F (2013). Spontaneous cervical artery dissection: the Borgess classification. Front Neurol.

[19] Lyrer P, Engelter S (2010). Antithrombotic drugs for carotid artery dissection. Cochrane Database Syst Rev.

[20] Limaye K, Abla AA (2015). We will use antiplatelets as our first choice for prevention of stroke recurrence in Cervical Arterial Dissection After Reading CADISS--will you?. World Neurosurg.

[21] Traenka C, Jung S, Gralla J (2018). Endovascular therapy versus intravenous thrombolysis in cervical artery dissection ischemic stroke – Results from the SWISS registry. Eur Stroke J..

[22] Bernardo F, Nannoni S, Strambo D, Bartolini B, Michel P, Sirimarco G (2019). Intravenous thrombolysis in acute ischemic stroke due to intracranial artery dissection: a single-center case series and a review of literature. J Thromb Thrombolysis.

[23] Spanos K, Karathanos C, Stamoulis K, Giannoukas AD (2016). Endovascular treatment of traumatic internal carotid artery pseudoaneurysm. Injury.

[24] Engelter ST, Traenka C, Lyrer P (2017). Dissection of cervical and cerebral arteries. Curr Neurol Neurosci Rep.

